# ELDER MISTREATMENT RISK FACTORS AMONG HOME HEALTH CARE SERVICE RECIPIENTS

**DOI:** 10.1093/geroni/igae098.2923

**Published:** 2024-12-31

**Authors:** Karen Schlag, Monique Pappadis, Jordan Westra, Yong-Fang Kuo, Leila Wood, Rebecca Czyz, Mukaila Raji, Charles Mouton

**Affiliations:** The University of Texas Medical Branch, Galveston, Texas, United States; The University of Texas Medical Branch, Galveston, Texas, United States; The University of Texas Medical Branch, Galveston, Texas, United States; University of Texas Medical Branch, Galveston, Texas, United States; The University of Texas Health Science Center, Houston, Texas, United States; The University of Texas Medical Branch, Galveston, Texas, United States; University of Texas Medical Branch, Galveston, Galveston, Texas, United States; The University of Texas Medical Branch, Galveston, Texas, United States

## Abstract

Elder mistreatment (EM) research has often focused on EM risk factors among older adults in nursing homes and less on those homebound in Medicare-based home health care (HHC). We aimed to apply and improve a Medicare claims-based EM risk assessment model previously developed for primary care settings using the Outcomes and Assessments Information Set (OASIS) data in addition to Medicare claims. Drawing from the 20% national Medicare data, beneficiaries aged 66 and older receiving HHC in 2016, without an EM diagnosis in 2015 or 2016, and Medicare Part A/B/D eligible with no HMO through 2018 were included (N=159,278). Logistic regression models predicting 2-year EM diagnosis were evaluated. The cohort was split into training (50%), testing (25%), and validation (25%) samples. Risk factors from claims (e.g., demographics, health conditions, frailty scores (0-1), medical screening/procedure codes, and social determinants of health) and the OASIS (e.g., cognition, self-care, mobility, behavioral problems, living situation, and readmission risk score) were included. EM diagnosis prevalence was 0.4%. Our models’ discriminatory power, c-statistic, of the claims-based model and the claims-based model with HHC assessments was 71.2 and 76.2, respectively. Medicare dual eligibility, disability entitlement, certain medical conditions (e.g., hepatitis, learning disabilities, post-traumatic stress disorder, COPD), and housing and income problems were associated with increased risk of EM in both models. OASIS data showed that patients living alone and with greater disruptive behaviors were at increased EM risk. Including assessment data can improve the claims-based algorithm to predict EM among HHC recipients.

